# Complete Genome Sequence of a Novel Satellite Virus Associated with Cassava Plants

**DOI:** 10.1128/genomeA.00164-17

**Published:** 2017-04-20

**Authors:** Adriana N. Souza, Fábio N. Silva, Claudine M. Carvalho

**Affiliations:** a Universidade Federal de Viçosa, Departamento de Fitopatologia, Viçosa, Minas Gerais, Brazil; b Universidade do Estado de Santa Catarina, Lages, Santa Catarina, Brazil

## Abstract

A novel satellite virus of 1,228 bp in length was found in a single cassava plant. Bioinformatic analyses show that it has two open reading frames (ORFs) in its genome, probably encoding a coat protein of 156 and a putative protein of 90 amino acids.

## GENOME ANNOUNCEMENT

Several plant viruses have subviral agents associated with them, for example, the satellite viruses: these are RNA molecules with less than 1,500 nucleotides that encode their own coat proteins (CP) and are dependent on their helper virus for replication, since they do not encode their own polymerase (1). In order to detect, identify, and characterize new viral agents associated with cassava in Brazil, dsRNA was extracted from a cassava plant, following the protocol developed by Valverde et al. (2), and submitted to deep sequencing analysis. A total of 30 g of tuber tissue from the phloem region was submitted to dsRNA isolation through cellulose chromatography. A total of 10 µg of dsRNA was sent for next-generation sequencing on the 454 GS-FLX Plus Platform (Macrogen, South Korea). All of the reads obtained in the sequencing were assembled using the software Geneious 7.1.5 (3). The assembled contigs were submitted to BLAST analysis (https://blast.ncbi.nlm.nih.gov/Blast.cgi) in order to identify these sequences. For unknown sequences, ORF Finder analysis (https://www.ncbi.nlm.nih.gov/orffinder/) was also conducted to identify any proteins showing some similarity with viral proteins.

A total of 126,433 reads were obtained through deep sequencing. After assembly of the reads, 666 contigs were obtained and submitted to nucleotide BLAST analysis. Most contigs showed nucleotide identity with plant genes and a few contigs showed no identity with sequences in the National Center for Biotechnology Information (NCBI) database. These unknown sequences were submitted to ORF Finder analysis, and the putative open reading frames (ORFs) found were submitted to protein BLAST analysis, in an attempt to identify putative proteins encoded by them. Just one of the total contigs obtained showed similarity with viral genomes. This contig was generated by 10 reads and had 946 nt in length, presenting two putative ORFs, both with their highest amino acid identities with grapevine satellite virus proteins (4).

To obtain and confirm the complete nucleotide sequence of the new satellite, primers were designed based on the sequence obtained by deep sequencing to amplify the 5′ ends of its plus and minus strands, using a 5′RACE system for rapid amplification of cDNA ends (Invitrogen). Amplified fragments were purified, linked into pGEM-T Easy Vector (Promega, USA), transformed into *Escherichia coli* DH5α, and sequenced. The sequence revealed a complete genome of 1,228 bp in length, with C+G content of 40.1% and two putative ORFs. The first ORF starts at nucleotide 179 and finishes at nucleotide 649. This ORF probably encodes a protein with 156 amino acids and has 57% of amino acid identity with ORF1 of grapevine satellite virus (4). This protein also presents a conserved domain from the *Potexvirus* coat superfamily. The second ORF starts at nucleotide 646, overlapping the last four nucleotides of the first ORF, and finishes at nucleotide 918, probably encoding a protein with 90 amino acids. This protein showed 38% of amino acid identity with ORF2 of grapevine satellite virus (4). A second start codon is present at nucleotide 709. This RNA molecule was denominated cassava satellite virus.

### Accession number(s).

The complete genome sequence of the cassava satellite virus isolate has been deposited at GenBank under the accession no. KY607769.
